# Delta activity independent of its activity as a ligand of Notch

**DOI:** 10.1186/1471-213X-5-6

**Published:** 2005-03-10

**Authors:** Lee-Peng Mok, Tielin Qin, Boris Bardot, Matthew LeComte, Asal Homayouni, Francois Ahimou, Cedric Wesley

**Affiliations:** 1 Department of Microbiology and Molecular Genetics, 322 Stafford Hall, 95 Carrigan Drive, The University of Vermont, Burlington, VT 05405, USA; 2 Rollins School of Public Health, Emory University, Atlanta, GA 30322, USA; 3 INSERM E365, Faculte de Medecine Lariboisiere, 10 avenue de Verdun, Paris 75010, France; 4 College of Veterinary Medicine, Kansas State University, Manhattan, KS 66506, USA; 5 Department of Civil Engineering, The University of Minnesota, Minneapolis, MN 55455, USA

## Abstract

**Background:**

Delta, Notch, and Scabrous often function together to make different cell types and refine tissue patterns during Drosophila development. Delta is known as the ligand that triggers Notch receptor activity. Scabrous is known to bind Notch and promote Notch activity in response to Delta. It is not known if Scabrous binds Delta or Delta has activity other than its activity as a ligand of Notch. It is very difficult to clearly determine this binding or activity *in vivo *as all Notch, Delta, and Scabrous activities are required simultaneously or successively in an inter-dependent manner.

**Results:**

Using Drosophila cultured cells we show that the full length Delta promotes accumulation of Daughterless protein, *fringe *RNA, and *pangolin *RNA in the absence of Scabrous or Notch. Scabrous binds Delta and suppresses this activity even though it increases the level of the Delta intracellular domain. We also show that Scabrous can promote Notch receptor activity, in the absence of Delta.

**Conclusion:**

Delta has activity that is independent of its activity as a ligand of Notch. Scabrous suppresses this Delta activity. Scabrous also promotes Notch activity that is dependent on Delta's ligand activity. Thus, Notch, Delta, and Scabrous might function in complex combinatorial or mutually exclusive interactions during development. The data reported here will be of significant help in understanding these interactions *in vivo*.

## Background

Notch (N) and Delta (Dl) are cell surface proteins that are required for differentiation of almost all tissues in the fruit fly *Drosophila melanogaster*. They are evolutionarily conserved, functioning similarly in animals from worms to humans [1,2]. The best-known instance of their function is the process of lateral inhibition that initiates differentiation of the neuronal and epidermal tissues from proneural cells that are predisposed to making the neuronal tissue. Proneural cells express high levels of the neuronal transcription co-factors from the Achaete Scute Complex (ASC) or related genes [3,4]. These factors require their partner Daughterless (Da) to activate transcription of the neurogenesis genes [5-7]. Da is expressed at low levels in all Drosophila cells [8] and up regulated in proneural cells specified to differentiate the neurons [5]. Whether or not the up regulation of Da expression is part of lateral inhibition is not clear in Drosophila. In *Caenorhabditis elegans*, however, the differential accumulation of the Da homolog HLH-2 is the earliest detectable difference between the cells taking up alternate fates during lateral inhibition [9]. As N and Dl are known to regulate Da expression [10], it is very possible that Da expression is regulated during lateral inhibition in flies as well.

When N expressed on one proneural cell binds Dl expressed on the neighboring proneural cell, N is proteolytically cleaved to release the Notch intracellular domain (N^intra^) from the plasma membrane. N^intra ^translocates to the nucleus and, in association with the transcription factor Suppressor of Hairless (SuH), activates transcription of the *Enhancer of split Complex *(E(spl)C) genes. Cells that express a high level of *E(spl)C *RNA suppress their neuronal predisposition, become the epidermal precursor cells (EPCs), and differentiate the epidermis. Cells that express a low level of *E(spl)C *RNA and a high level of Da protein become the Neuronal Precursor Cells (NPCs) and differentiate the nervous system [1,2,5,11]. From here onwards, we refer to this SuH dependent N activity that promotes expression of *E(spl)C *RNA as SuH/N^intra ^signaling. A 1.5 to 2-fold difference in the level of SuH/N^intra ^signaling is sufficient to initiate specification of the EPCs and the NPCs [11]. This difference is amplified by subsequent activities of N and Dl, or activities of other genes responding to the initial difference in the level of SuH/N^intra ^signaling. The lateral inhibition process described above is repeatedly used during development for differentiation of various tissues with minor variations or changes in target genes.

Scabrous (Sca) is a secreted factor that is produced at high levels in the NPCs and functions non-autonomously to promote specification of the EPCs during differentiation of the compound eye and the bristle organ [12,13]. In its absence, lateral inhibition is not abolished but is reduced in strength or becomes imprecise indicating that Sca only refines the process. Sca binds N and stabilizes it. These actions promote formation of sharp boundaries between neuronal and non-neuronal cells during development of the compound eye [14]. The possibility that Sca might bind Dl as well is suggested by the observation that simultaneous over expression of Sca almost completely blocks the effect of Dl over-expression on wing margin development but hardly modifies the effect of N over-expression [15]. Dl and Sca have also been observed to co-localize in intracellular vesicles in vivo [13]. The observations that Sca can promote N activity [14] but block Dl activity are paradoxical as SuH/N^intra ^signaling is very much dependent on the activities of both N and Dl. One explanation for this paradox could be, that Sca promotes lateral inhibition by having one effect through N and a different one through Dl. Therefore, we addressed the following questions in this study. Does Sca bind Dl? If yes, does it affect any Dl activity? Are there Dl activities independent of its activity as a ligand of N? Is Sca capable of activating N in the absence of Dl?

N and Dl are expressed in almost all cells *in vivo *and N receptor activities in response to Dl binding are widely used during development. In developmental instances where Sca is present, the expression data suggest that both N and Dl will have access to Sca. Thus, it is very difficult to separate *in vivo *the activities of N alone, Dl alone, N on Dl, Dl on N, Sca on N, Sca on Dl, and Sca on N and Dl together. Therefore, we addressed the questions posed above in an *in vitro *model system based on Drosophila Schneider (S2) cells. S2 cells do not express the endogenous N, Dl, or Sca [14,16]. S2 cells expressing N (S2-N cells) mixed with S2 cells expressing Dl (S2-Dl cells) reproduce all aspects of lateral inhibition [16-22]. Using these cells and the medium prepared from S2 cells expressing Sca into the medium [14], we show that Sca binds Dl, Dl has activity independent of its activity as a ligand of N, Sca can affect this activity of Dl, and Sca can activate N in the absence of Dl. These observations would be useful for undertaking the challenging task of determining how the various activities of N, Dl, and Sca are integrated during tissue differentiation.

## Results

### Sca associates with Dl

Although N and Sca complexes could be immuno-precipitated [14], we, and others [23], had failed to detect Sca on S2-N cells. We suspected that some factor present in the tissue culture medium was washed away when the cells were processed for immuno-fluorescent detection of Sca. To overcome such problems, we made Sca-GFP and established stable S2 cells expressing it (S2-Sca-GFP cells). S2-Sca-GFP cells produced the Sca-GFP protein of the expected size (as determined by western blotting) and both Sca and GFP antibodies recognized this protein (data not shown). We concluded that S2-Sca-GFP cells expressed the expected Sca-GFP protein and used the conditioned medium from these cells to treat live S2-N, S2-Dl, and S2 cells.

**Figure 1:**
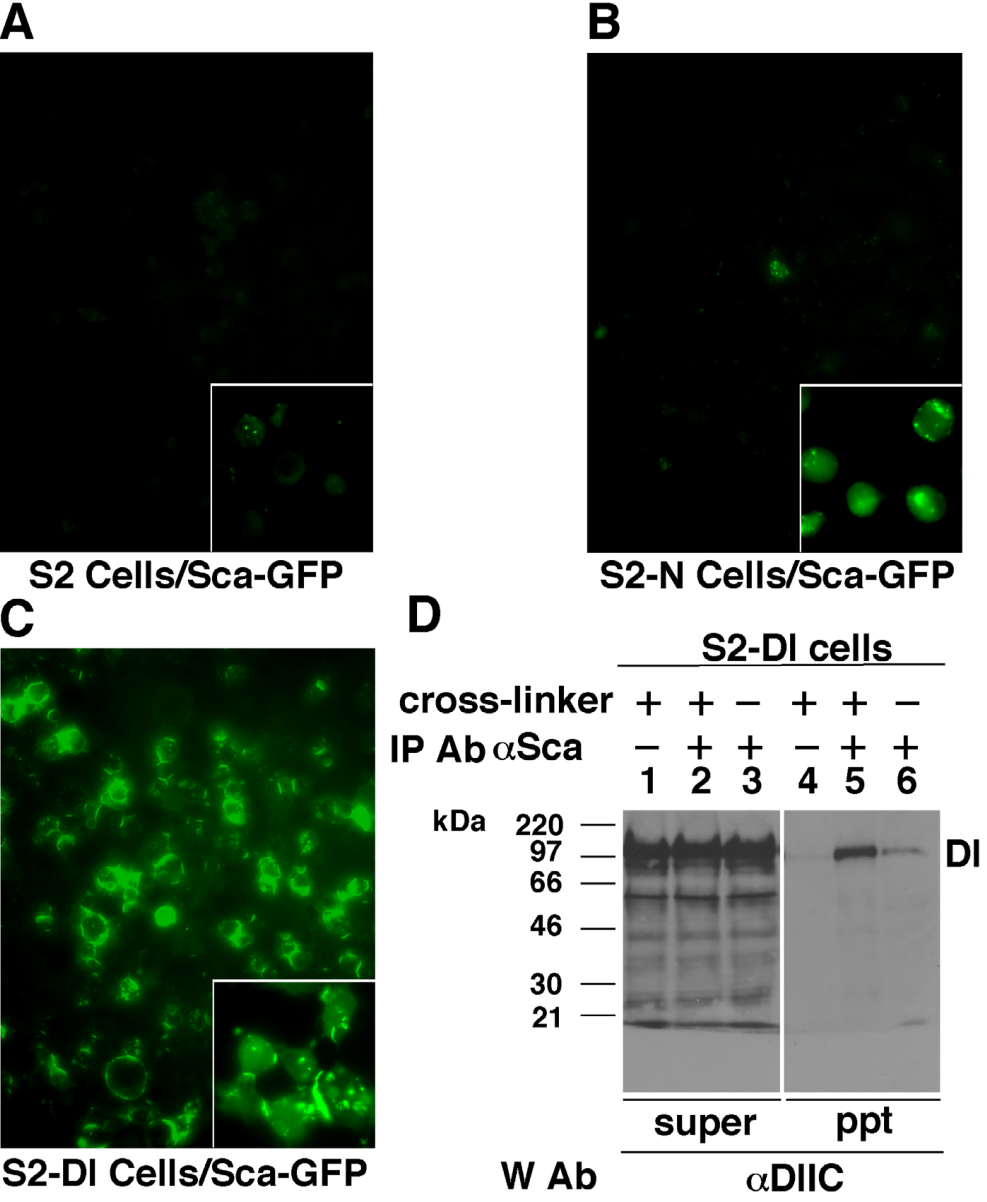
Sca associates with Dl. **A-C**. Fluorescent photomicrographs of different cell lines treated with Sca-GFP medium for 30 minutes. Cells simultaneously fixed and rinsed in 4% paraformaldehyde/1X PBS are shown in the insets. Experiments were repeated three times. **D**. Western blots showing recovery of Dl in Sca immuno-precipitates from total protein extracts prepared from S2-Dl cells treated with S2-Sca cells. S2-Sca cells were used instead of Sca conditioned medium to maximize the ratio of bound to unbound Sca. Cross-linker = membrane insoluble and cleavable 3,3'- Dithiobis (sulfosuccinimidylpropionate) (DTSSP), which cross-links proteins interacting at the cell surface. IP Ab = antibody used for immunoprecipitation; W Ab = antibody used on the western blot; ppt = immunoprecipitate; super = supernatant. Experiments were repeated two times.

Live S2-Dl cells showed the strongest GFP signals, followed by live S2-N cells, and then live S2 cells (Fig. 1A–C). The signals were so strong on the S2-Dl cells that the signals on S2-N cells were not obvious at the same brightness/contrast settings. When cells were simultaneously fixed and rinsed with 1X PBS, the signals were comparable at the same settings (insets in Fig. 1A–C). Signals could not be detected on S2-N or S2-Dl cells after three 5-minute washes with 1X PBS, confirming our suspicion that the standard immuno-fluorescence procedure is inappropriate for detecting Sca binding on S2-N or S2-Dl cells. Secreted GFP did not bind the surfaces of any of these cells (data not shown). This indicated that the Sca part of Sca-GFP fusion protein bound the S2-N and S2-Dl cell surfaces. In all experiments conducted to determine the activity of Sca, N, or Dl, that are described below, we used only S2 cells expressing the wild type Sca because (1) we do not perform washes to remove non-specifically bound proteins and (2) we wanted to avoid possible GFP associated effects (stability, etc.).

We have previously shown that Sca and N form complexes [14]. To determine whether Sca forms complexes with Dl, we performed immuno-precipitation experiments with S2-Dl cells that were co-cultured with S2-Sca cells. Proteins interacting at the cell surfaces were either cross-linked or un-linked prior to cell lysis for protein extraction. Membrane insoluble cross-linkers improve recovery of cell surface complexes [18,24]. Sca immuno-precipitation recovered Dl strongly in the presence of cross-linkers and relatively weakly in the absence of cross-linkers (Fig. 1D). No bands were observed when S2 cells were used instead of S2-Dl cells (data not shown). In the reverse experiments, Dl immuno-precipitations failed to recover Sca, possibly because there was too much unbound Dl in the extracts. Dl and Sca were not detected in the absence of immuno-precipitation antibodies (Fig. 1D, lanes 1 and 4) or in the absence of Scabrous (data not shown). We also recovered Sca in Dl immuno-precipitations and Dl in Sca immuno-precipitations from protein extracts of wildtype embryos (data not shown). These observations indicated that Sca associates with Dl. We explored the consequence of this association.

### Da expression in Dl cells is reduced in response to Sca

**Figure 2:**
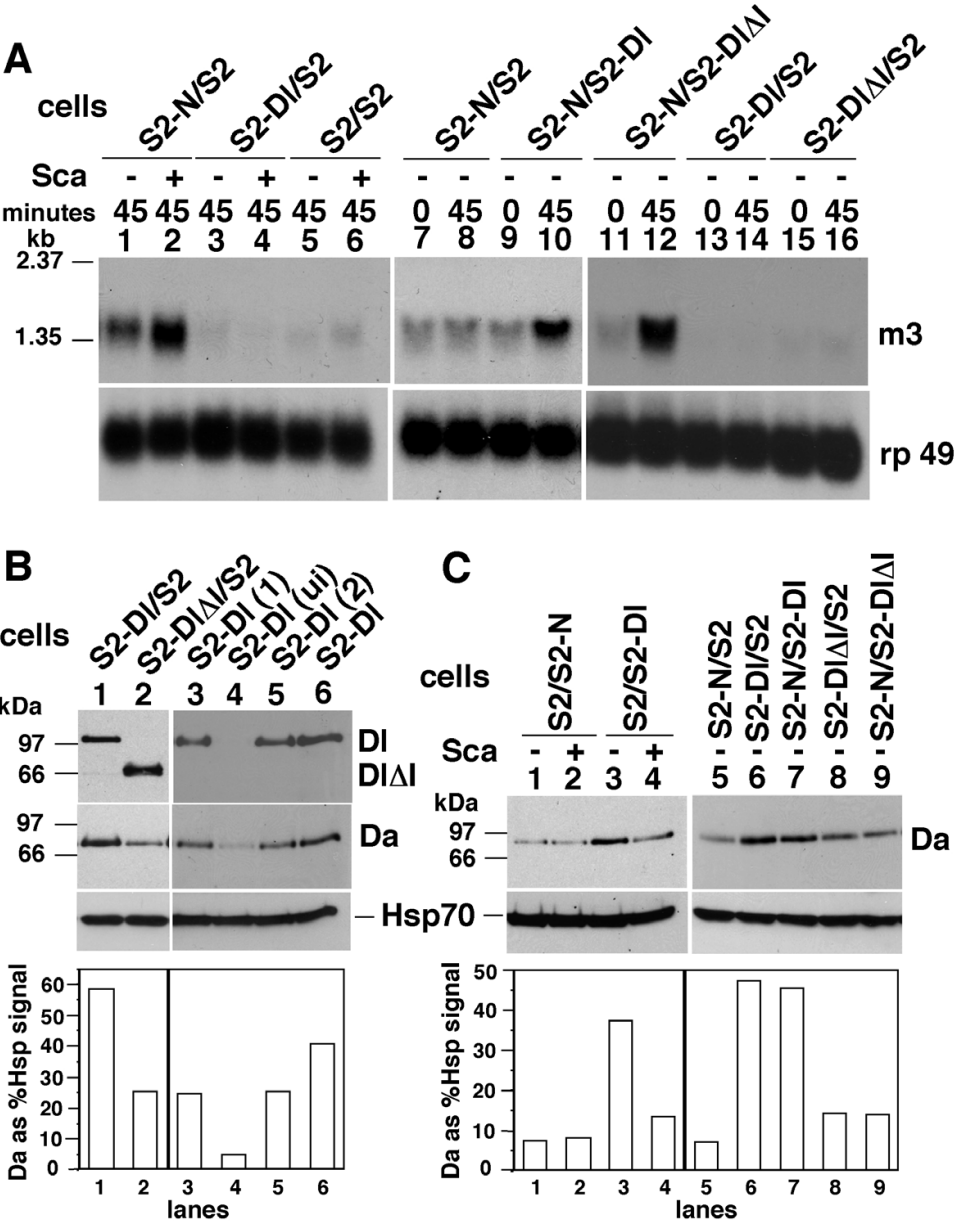
Dl down-regulates Daughterless protein expression, and N up-regulates *E(spl)C m3 *gene expression, in response to Scabrous. **A**. Northern blots of total RNA from the indicated cell mixtures extracted at 0 or 45 minutes after treatment with medium containing Sca (+) or not (-). Gene probes used are shown on the right. m3 = *E(spl)C m3 *and rp 49 = a ribosomal protein gene used to show the levels of total RNA in the lanes in all northern blots. Sca = conditioned medium prepared from the S2-Sca stable cell line in all experiments here onwards. The control medium used along side Sca medium (-) was prepared from heat shocked S2 cells. Experiments were repeated two times. For unknown reasons, the medium collected from heat shocked S2 cells (used in lanes 1, 3, and 5) produced higher background levels of *E(spl)C m3 *RNA in S2-N cells (lane 1). **B**. Western blots showing the levels of Da and Dl in different Dl cell lines. S2-Dl, S2-Dl(1) and S2-Dl(2) are independently established hsDl cell lines. Ui = un-induced (i.e., not heat shocked). Hsp70 = heat shock 70 protein used to show the levels of proteins in the lanes of all western blots. Dl and DlΔI were detected with αDlEC. Da signals here (and the indicated signals elsewhere) were quantified relative to Hsp70 (western blots), rp49 (northern blots), or other indicated molecules, using the NIH Image 1.63 program. These experiments were repeated more than ten times. **C**. Western blots showing Da levels in the indicated cell mixtures, with (+) or without (-) Sca. These experiments were repeated five times.

N promotes expression of *E(spl)C m3 *gene in response to Dl [19,20]. We examined whether Sca promoted expression of *E(spl)C m3 *in S-N cells or S2-Dl cells and found that it was indeed the case with S2-N cells, but not with S2-Dl cells (Fig. 2A, lanes 1–6). S2-N cells showed a low level of *E(spl)C m3 *expression when S2-Dl or S2-DlΔI cells were replaced with S2 cells, in the absence of Sca (Fig. 2A, lanes 1, 7–8); S2-Dl or S2-DlΔI cells mixed with S2 cells did not show any accumulation (Fig. 2A, lanes 13–16). The low level of *E(spl)C m3 *RNA expression in S2-N cells in the absence of ligands is due to the low level of N^intra ^produced upon induction of N expression in S2 cells [18]. This expression increases upon ligand treatment [18], resulting in increased expression of *E(spl)C m3 *RNA expression (Fig. 2A, lanes 2, 10, 12). Numerous repetitions of the experiments indicated that Dl is a more potent ligand of N than Sca with respect to induction of *E(spl)C m3 *expression (data not shown).

*E(spl)C m3 *expression appeared to be solely dependent on N activation and the Notch intracellular domain as it was promoted in S2-N cells treated with either S2-Dl cells or S2-DlΔI cells (Fig. 2A, lanes 7–12). As DlΔI lacks the intracellular domain, it is expected to behave only as a ligand of N and not generate any intracellular signal of its own in response to N binding. We observe comparable levels of SuH/N^intra ^signaling with S2-Dl and S2-DlΔI cells (Fig. 2A, lanes 10, 12). This is not consistent with the *in vivo *findings that the Dl intracellular domain (lacking in DlΔI) is required for SuH/N^intra ^signaling, possibly for promoting Dl internalization that results in exerting a 'pull' on N and increased production of N^intra ^[21,25-29]. However, our results are consistent with other S2 cell studies showing that even fixed S2-Dl cells can promote production of SuH/N^intra ^signaling in S2-N cells [19]. Thus, it is possible that that Dl internalization and pulling is not required for SuH/N^intra ^signaling in S2 cells. In any case, in our S2 cell system, the S2-N and S2-Dl cells require shaking for formation of cell aggregates. As a consequence, we shake all cell mixtures, including those containing the secreted ligand Sca. This shaking might have simulated the pulling effect and overcome any deficiency DlΔI might have in this regard thereby resulting in a level of SuH/N^intra ^signaling that is comparable to that produced by the full length Dl.

We examined the expression of various proteins known to be involved in lateral inhibition to find out if Dl expression affected them. They were Numb, Dishevelled, Suppressor of Hairless, Wingless, Hairless, Hairy, Achaete, Da, and Armadillo. We found a relatively high level of Da protein in S2-Dl cells compared with the level in S2-DlΔI cells (Fig. 2B, lanes 1–2). Similar levels of Da were expressed in S2-DlΔI and S2 cells (data not shown). Two independently transfected S2-Dl cell lines also showed high levels of Da, and un-induced S2-Dl cells showed background levels of Da, indicating that Dl expression promotes Da expression (Fig. 2B, lanes 3–6). Increase in Da levels appeared to be specifically linked to Dl expression, as S2-N cells did not show an increase (Fig. 2C, compare lanes 1 & 3). Overall, Da expression in S2-Dl cells was 2.18X higher (+/- 0.37, p < 0.05) than the level in S2 cells, sometimes more than 5X higher. Here and in all cases to follow, the blots shown in the figures are the most representative blots among replications. Graphs show quantification, relative to standards or other proteins (as indicated), of signals on the blots composing the figures as the response can be assessed only in comparison to the control lanes in the same experiment. Pooling data from all replications of an experiment obscured the response, or misrepresented the data, due to variation between different batches of cells. Therefore, we computed error variance for the degree of response over all replications of an experiment. These values for important responses are mentioned in the text. The number of repetitions of an experiment is indicated in the figure legends.

We examined Da levels in S2-Dl and S2-N cells that were treated or not treated with Sca conditioned medium. We found that Sca treatment decreased the levels of Da in S2-Dl cells (Fig. 2C, lanes 3–4). The levels in S2-N cells were low and unaffected by Sca treatment (Fig. 2C, lanes 1–2). These experiments suggested that Sca blocks accumulation of Da in S2-Dl cells (2.81X, +/- 0.59, p < 0.05). We also determined the levels of Da when S2-N and S2-Dl cells were together in the absence of Sca. The level of Da never increased (Fig. 2C, lanes 6–7). As N activation suppresses *daughterless *RNA expression [18], it was possible that N activation suppressed Da expression in S2-N cells and masked an increase in S2-Dl cells. To determine if this was the case, we compared the level of Da in mixtures of S2-DlΔI cells and S2 cells with mixtures of S2-DlΔI cells and S2-N cells. As S2-Dl and S2-DlΔI cells activate N equally well (see Fig. 2A, lanes 9–12), any change in Da level would be due to N activation. We found comparable levels of Da in the two samples (Fig 2C, lanes 8–9). Thus, S2-Dl cells do not appear to increase Da expression in response to S2-N cells.

When S2-N and S2-Dl cells were together in the presence of Sca, the levels of Da protein and *E(spl)C m3 *RNA were very variable (data not shown). This was possibly due to the varying combinations of Sca effect on S2-Dl cells, Sca effect on S2-N cells, Dl effect on S2-N cells, and N effect on S2-Dl cells.

**Figure 3:**
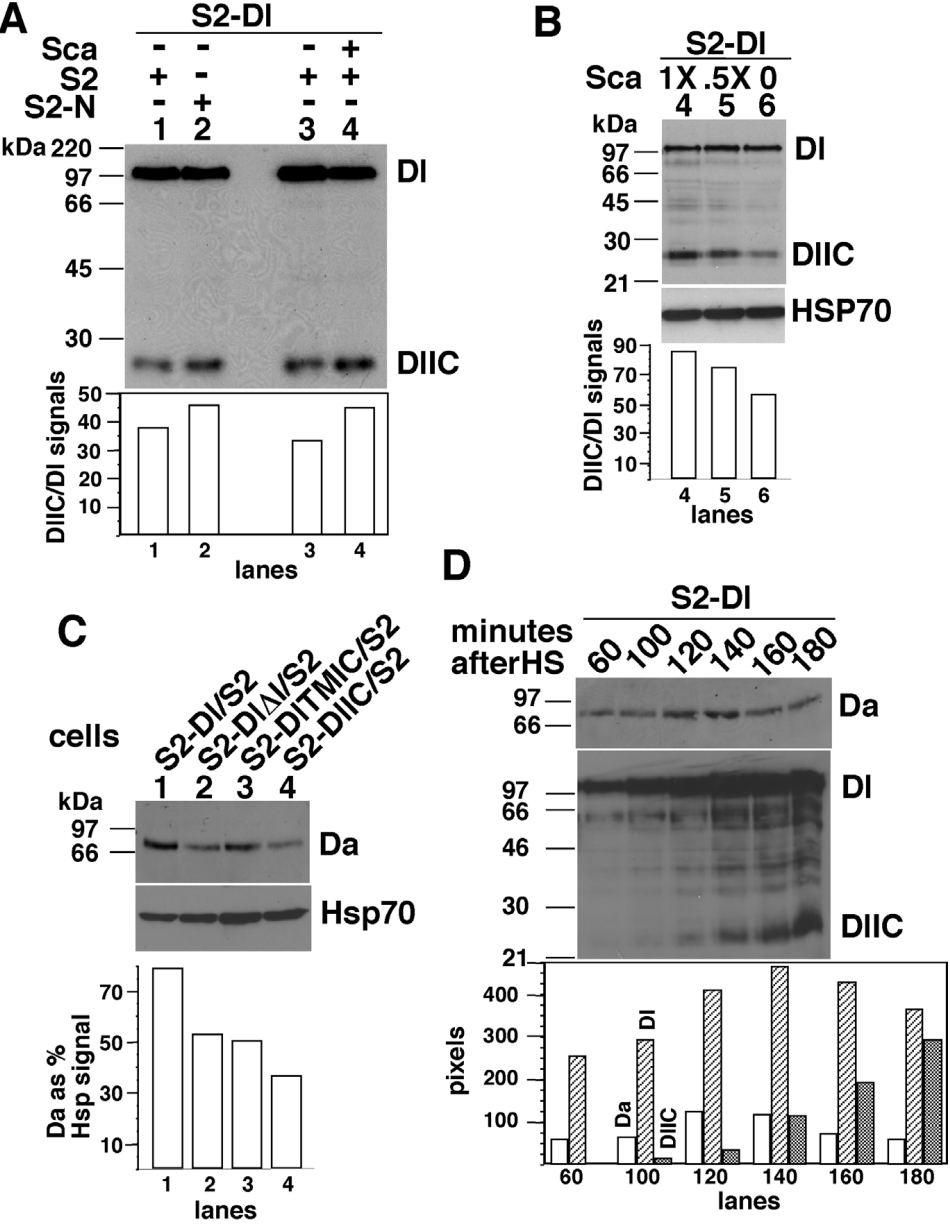
The levels of cleaved Dl intracellular domain is not associated with high levels of Da. **A**. Western blots (from a 8% SDS-PAGE) showing the level of Dl and DlIC in the indicated cell mixtures, with (+) or without (-) Sca. **B**. Western blots (from a 12% gel) showing the levels of Dl and DlIC in S2-Dl cells treated medium containing different levels of Sca. **C**. Western blots showing the levels of Da in the indicated cell mixtures. **D**. Western blots showing the levels of Da, Dl, and DlIC at different times following heat shock induction of Dl in S2-Dl cells. All experiments were repeated at least three times.

Dl is processed to produce Dl intracellular domain (DlIC), constitutively, and the levels of DlIC increase upon N treatment [30-33]. Therefore, we examined the levels of DlIC following treatment of S2-Dl cells with S2-N cells or Sca medium. We found that the DlIC levels increased by 25 to 50% (relative to Dl levels) with both treatments (Fig. 3A and 3B). We examined the levels of Da in S2 cells expressing DlIC and DlTMIC (lacking the extracellular domain only and including the transmembrane domain). The levels of Da in S2-DlIC and S2-DlTMIC were always comparable to or lower than the level in S2 or S2-DlΔI cells (Fig. 3C). Also, we found Da levels to be negatively correlated with the accumulation of DlIC in time-course experiments (Fig. 3D). This negative correlation could be a direct consequence of the accumulation of DlIC or due to autoregulation of the *da *gene [34]. We examined the levels of Da in flies expressing heat shock induced Dl, N, or Sca, in flies heterozygous for the null alleles of N or Dl, and in flies homozygous for a null allele of Sca. We found that Da expression was strongly associated with Dl expression rather than with N expression, and inconsistently associated with Sca expression. These data are consistent with our findings in S2 cells but are not shown, as we cannot clearly separate the effects of N, Dl, and Sca, the way we can do in S2 cells. The experiments described in this section indicate that Da accumulation is promoted by the full-length Dl, not by the Dl intracellular domains (DlIC or DlTMIC), and Sca suppresses the activity of the full length Dl. The experiments also indicate that Sca promotes *E(spl)C m3 *RNA expression in S2-N cells even in the absence of Dl.

### Dl regulates expression of *fringe *and *pangolin*

To gather additional evidence for Dl activity independent of its activity as a ligand of Notch, we performed microarray experiments using the Affymetrix Drosophila GeneChip Arrays to compare gene expression in S2 cells and S2-Dl cells. Many genes relevant to known Dl functions responded in S2-Dl cells (at p < 0.05, n = 3 × 2 pooled samples): axonal path finding genes (e.g., Gef64C, 39.38X,Up; Tenascin major, 6.77XUp), actin-based cell motility and kinases (Rho-Kinase, 15.08XUp; Rhophilin 3.4XUp; nemo 1.72XUp, basket 1.69XUp; pointed 2.2XUp), N signaling pathway genes (e.g., reaper, 2.26XUp; sanpodo, 1.91XUp), and oogenesis genes (e.g., swallow, 8.12XUp; sprouty, 3.58Xup). Expression of *transformer*, was also up (1.76X) and it is significant in the light of our observation that Dl promotes expression of Da: both Da and *transformer *are involved in sex determination. Expression of *da *RNA was not significantly increased in S2-Dl cells, possibly due to the negative part of the *da *gene autoregulation system [34]. The detailed analyses with validations will be published elsewhere. The experiment also identified *fringe *(*fng*) and *pangolin *(*pan*) as responding to Dl expression. *fng *is a glycosyl transferase that regulates the affinity of N for Dl [35-37], and possibly also the affinity of Dl for N [38]. *pan *is a transcription factor functioning in the Wingless (Wg) pathway [39,40]. Notch and Wg pathways interact closely at many differentiation events during development [24,41-44]. Therefore, we chose *fng *and *pan *for further investigation.

**Figure 4:**
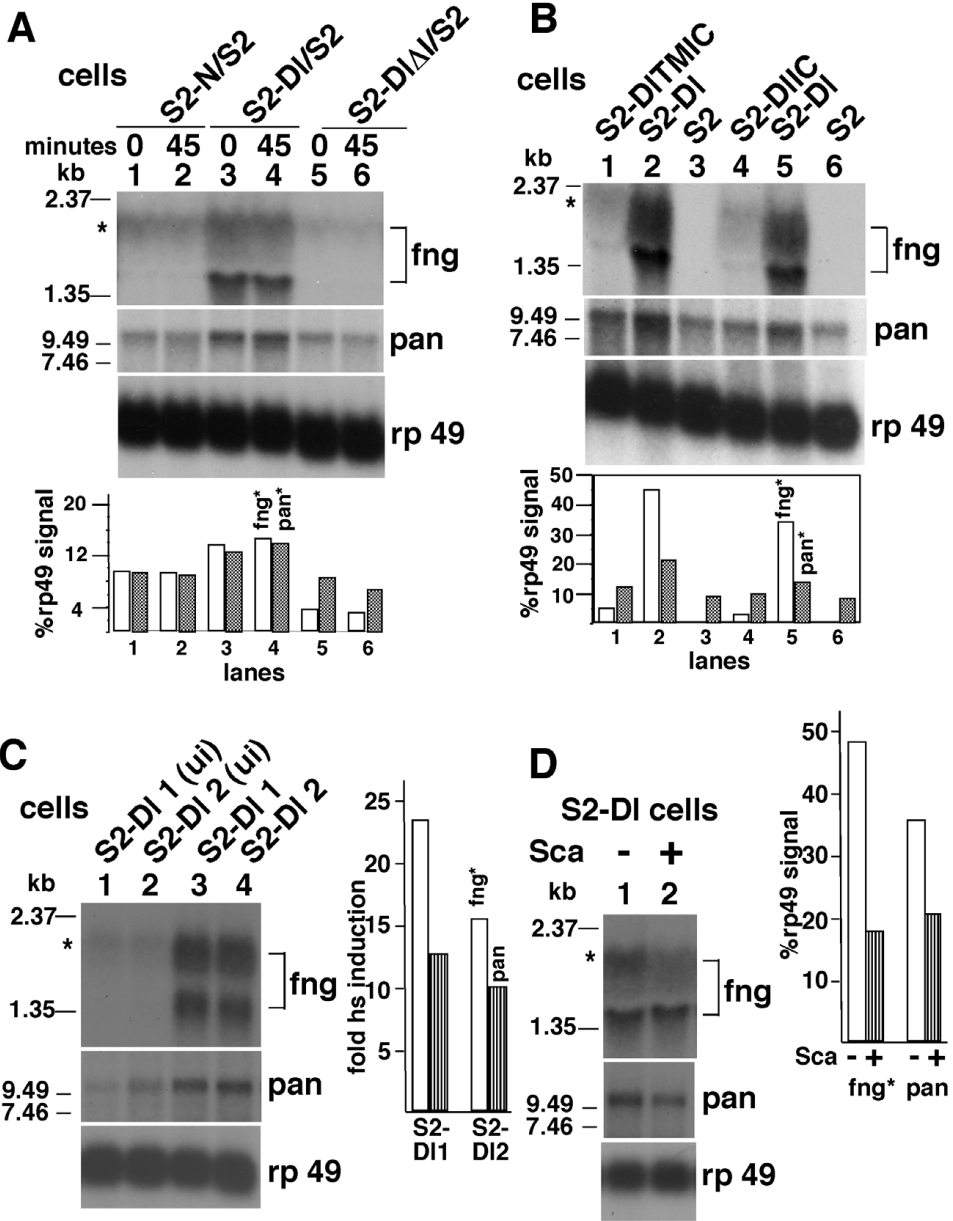
Dl promotes expression of *fng *and *pan*. **A**. Northern blots showing *fng *and *pan *expression in the indicated cell mixtures at 0 and 45 minutes after cell mixing. **B**. Northern blots showing *fng *and *pan *expression in the indicated cell lines two hours after induction of expression. **C**. Northern blots showing *fng *and *pan *expression in two other independently established S2-Dl cell lines. Cells used for lanes 1–2 were uninduced (ui); cells used for lanes 3–4 were heat shock induced. **D**. Northern blots showing *fng *and *pan *expression in S2-Dl cells that were either untreated or treated with Sca medium. All experiments were repeated at least three times. The *fng *band marked with an asterisk corresponds to the published mRNA [35]. Only this band was used for *fng *quantification. The *pan *band shown is consistent with the information described in van de Wetering et al. [40] and Brunner et al. [39].

Northern blot analyses showed that the expression of *fng *and *pan *was higher in S2-Dl cells compared with S2-N or S2-DlΔI cells (Fig. 4A). DlIC and DlTMIC promoted expression of *pan *and *fng *weakly, if at all (Fig. 4B). Two independently established S2-Dl cell lines also showed higher levels of *fng *and *pan *RNAs (Fig. 4C). Sca treatment S2-Dl cells reduced the levels of *fng *and *pan *RNA (Fig. 4D). This reduction was expected as Sca reduces the levels of the full length Dl (see Fig. 3). Thus, just as it was the case with Da expression, the full length Dl, not any of its parts, strongly promoted *pan *and *fng *expression. We examined the levels of *fng *and *pan *RNA in flies expressing heat shock induced Dl, N, or Sca, in flies heterozygous for the null alleles of N or Dl, and in flies homozygous for a null allele of Sca. We found that *fng *and *pan *RNA expression was strongly associated with Dl expression rather than with N expression, and inconsistently associated with Sca expression. These data are consistent with our S2-Dl cells data but are not shown, as we cannot clearly separate the effects of N, Dl, and Sca, the way we can do in S2 cells.

## Discussion

Our experiment addressed four questions. Does Sca bind Dl? If yes, does it affect any Dl activity? Are there Dl activities independent of its activity as a ligand of N? Is Sca capable of activating N in the absence of Dl? Results described in Figure 1 show that Sca binds Dl. This binding is not dependent on N as S2-Dl cells do not express N. We have previously shown that Sca binds N [14]. It is possible that Sca binds N or Dl stronger when they are present together on the same cell or on neighboring cells. It would be possible to test this in the future using Atomic Force Microscopy that is best suited for determining binding strengths of cell surface proteins like N or Dl [21]. Results in Figure 2A show that Sca can promote SuH/N^intra ^signaling through N in the absence of Dl, as S2-N cells do not express Dl. However, numerous repetitions of the experiment indicate that Sca is not as potent as Dl in this regard. This is consistent with the fact that lateral inhibition is blocked in the absence of zygotic Dl, which does not affect proneural cluster formation and thereby Sca expression. It would have been relatively easy to determine if over-expression of Sca in the absence of Dl rescues SuH/N^intra ^signaling phenotypes, and the extent of this rescue, if Dl did not have any activity independent of N. Hopefully, it would be possible in the future, when we better understand this Dl activity and are able to circumvent it. Results in Figure 2A also show that the expression of E(spl)C m3 gene, a target of SuH/N^intra ^signaling pathway, is responsive only to N indicating that this pathway is unlikely to be involved in mediating Dl activities.

Results described in Figures 2, 3, 4 and our microarray analysis show that Dl has activity independent of its activity as a ligand of N and Dl could be a receptor of Sca. This is clearly shown in experiments with S2-Dl cells that do not express N and we do not provide either N or Sca (Fig. 2B, C; 4A). The Dl activities we have described- promotion of expression of Da protein, *fng *RNA, and *pan *RNA- can be detected *in vivo *as well although the interpretation here is not simple due to the many possible interactions among N, Dl, and Sca that cannot be easily sorted out. However, these data (which we do not show) strongly suggest that the Dl activities we have described in S2 cells represent the *in vivo *Dl activities during development.

The N independent Dl activity we have described is dependent on the full length Dl, not just on its intracellular domain or the extracellular domain (Figs. 2C; 3; 5A-B). This is different from the situation with N whose activity is based on the activity of its intracellular domain [45-47]. Accordingly, treatment with Sca, which promotes production of the Dl intracellular domain, suppresses Dl activity related to Da rather than promote it (Figs. 2C; 3). This observation also indicates that Sca is able to affect Dl activities. A clean dissection of Sca effects through N from its effects through Dl would require identification of Sca binding sites on Dl, and N and Dl binding sites on Sca. We know that Dl and Sca bind different regions of N [14,48,49]. It would not be too surprising if Dl bound N and Sca in different regions, and if Sca bound N and D in different regions. With that knowledge and suitable mutants, we might be able to determine whether N, Dl, and Sca activities function in a mutually exclusive or combinatorial manner *in vivo*.

Dl activity that is independent of its N ligand activity has been speculated for some time. Efforts to identify it have intensified since the discovery that Dl gets proteolytically processed in the same manner as N [30-33]. However, it is extremely difficult to separate these two activities of Dl. The proof that the Dl activity we have identified actually functions during development in the expected manner, the details of the mechanisms underlying this function, and a better integration of the known functions of N, Dl, and Sca, will have to await more work which is neither quick nor simple. We hope that this work stimulates more efforts towards this task and makes this task a bit easier by identifying the potential of Sca as a regulator of Dl activity and the possibility that the full length Dl might be important for Dl activity independent of N, or Sca. Sca could also serve as a great tool for *in vivo *dissection of Dl response to N, as Sca and N appear to have a similar effect on Dl. It is quite likely that our experiments did not pick up Dl receptor activity in response to N or Sca. In any case, the potential developmental significance of our findings is briefly discussed below.

Da is a widely expressed protein and cells requiring its function show only a modest increase in its levels [5,6,8] indicating that, just like N^intra^/SuH signaling, small changes in Da levels might be sufficient for initiating or augmenting NPC specification and promoting neuronal differentiation. Small changes in Da levels might also be imposed by the built-in autoregulation of the *da *gene [34]. According to the well-accepted lateral inhibition model in the field, Dl activity as a ligand of N is postulated to increase in the NPCs and N receptor activity in response to Dl is postulated to increase in the EPCs [11]. Accordingly, Dl expression has been observed to increase in the NPCs or their equivalent cell types in certain instances involving N and Dl functions [50,51]. Our data suggest that an increase in Da levels in these instances could be due to the accumulation of the full length Dl, not any its parts such as DlIC, DlΔI, or DlTMIC. The requirement for the intracellular and the extracellular domains to be linked might mean that we have detected Dl activity requiring Dl's presence at the membrane or in the cytoplasm. This is consistent with the report that the cellular transformation ability of Jagged 1, a mammalian Dl homolog, requires an intact protein containing both the extracellular and the intracellular domains [52]. It is possible that DlIC, in the nucleus [31], promotes other activity that is different from the one described here. It is also possible that Da, *fng*, or *pan *might not be the direct target of the full length Dl activity. Our microarray data indicate that many other genes (including some in the RAS or EGFR signaling pathways) are strongly up regulated in Dl expressing cells. It is possible that one of these genes is the primary target. It is also possible that Da, *fng*, or *pan *accumulation is significant only in the context of these other genes. We will have to await validation of other putative targets of Dl activity, and evaluation of their role in lateral inhibition or other activities involving N and/or Dl, to determine if Da, *fng*, or *pan *are typical or atypical targets of Dl activity.

N/Dl binding and SuH/N^intra ^signaling are strongly affected by the functions of glycosyl transferases such as *fng*. The possibility that Dl, and not N, regulates *fng *RNA expression might explain some of the very complex functions of these glycosyl transferases and the complex interactions between N and Dl during lateral inhibition. As N and Dl activities are known to strongly interact with the Wg signaling pathway, it is interesting that Dl promotes *pan *expression. It is possible that Dl activity independent of N accounts for some of the interactions between the N and the Wg pathways. So far, these interactions have been considered only from the perspective of N receptor activity.

Lastly, our data suggest interesting interactions among Dl, N, and Sca in instances of lateral inhibition and tissue differentiation when their functions overlap. The full length Dl promotes Da accumulation, not any of its parts that might result from processing in response to N or Sca binding. Thus, both the processed N and Dl might promote EPC specification- processed N through *E(spl)C *RNA and processed Dl through suppression of Da expression. Consequently, lateral inhibition might initiate with symmetrical actions of N and Dl promoting EPC specification in all proneural cells. Sca might boost N and Dl processing in the incipient EPCs while suppressing them or not affecting them in the incipient NPCs. Thus, it is possible that Sca or Sca-like molecule have a role in breaking the symmetrical actions of N and Dl during certain lateral inhibition instances. It is also possible that Sca mediates long range N signaling during differentiation of some other tissues, either alone or in association with Dl, as proposed by Renaud and Simpson [13]. By extending our results, it might be possible to develop strong hypotheses for testing *in vivo*, cleanly sort the different activities of N, Dl, and Sca, and understand the fascinating *in vivo *developmental mechanisms involving N, Dl, and Sca.

## Conclusion

Sca binds Dl and suppresses a Dl activity that is independent of Dl's activity as a ligand of N. This Dl activity requires the full length Dl and is not enhanced by expression of just the Dl intracellular domain, which is different from the mechanism underlying Notch activity. Da protein, *fng *RNA, and *pan *RNA responds positively to the N independent Dl activity we have discovered. These could be direct or indirect targets. Our microarray analysis has identified many more putative targets of N independent Dl activity that can be explored for a better understanding of the complex interactions among Dl, Sca, and N during Drosophila development.

## Methods

### DNA constructs

Sca-Gfp: The stop codon of *sca *was replaced with a glycine codon and fused in-frame with GFP to obtain Sca-GFP. A Bam HI-KpnI fragment containing this *sca *sequence was cloned into pEGFP vector (Clontech). The XbaI fragment containing Sca-GFP coding fragment was cloned into the pCaSpeR-hs vector. DlΔI: A stop codon and a XbaI restriction site was introduced after the transmembrane domain using PCR. The PCR product was checked for mutations and used to replace the BstEII-BcgI fragment in the Dl cDNA. An Eco RI-XbaI fragment from this construct (Dl amino acid 1 to 620) was cloned into the pCaSpeR-hs vector. DlIC: The Dl intracellular region (codon 619 to the stop codon 881) was PCR amplified, checked for errors, and cloned into the BglII-XbaI sites in the pCaSpeR-hs vector.

### Cell lines and conditioned medium

S2-N, S2-Dl, and S2-Sca cells have been previously described [14,20,49]. Other cell lines were established using the standard calcium phosphate transfection procedure and hygromycin selection. Conditioned medium was produced as described in Powell et al. [14], using serum-free or serum-containing Shields and Sang's M3 medium. For experiments, cells were heat shocked at 37°C for 30 minutes in a water bath, allowed to synthesize proteins for 2 hours, washed in culture medium without serum, mixed with the appropriate cell lines, and shaken gently in 14 ml falcon tubes for two hours or the indicated time. See Wesley and Mok [20] for more details.

### Immunoprecipitations, western blotting, northern blotting, RNA in situ, and protein staining

Procedures described in Lieber et al. [43], Wesley [24], Wesley and Saez [18], and Wesley and Mok [20] were followed. Eight per cent SDS-PAGE systems were used for western blotting, unless otherwise indicated; 1% formaldehyde-MOPS agarose system for northern blottings. *fringe *cDNA (from Dr. Ken Irvine), rp49 cDNA, and rt-PCR amplified *pangolin *cDNA were used to prepare probes for northern blots. Incubation times with ligands were two hours for western blots and 45 minutes for all northern blots; it was three hours for *fng *and *pan *northern blot showing the effect of Sca (Fig. 4D).

Antibodies: αSca (mAb sca1) and αDlEC (C594.9B) were obtained from the Developmental Studies Hybridoma Bank; αGFP (G-6539) and αHsp70 (H-5147) from Sigma; αDlIC (GPC2) from Dr. Marc Muskavitch, αDlIC (dC-19) from Santa Cruz Biotechnology, αDa (DAM 109-10) from Dr. Claire Cronmiller; and αNI from Dr. Toby Lieber.

### Microarray analysis

Heat shocked S2 and S2-Dl cells were treated with Sca or non-Sca medium for 45 minutes before extracting RNA. GeneChip Drosophila Genome Arrays from Affymetrix were used. RNAs were extracted, checked, and processed for hybridization according to procedures suggested by Affymetrix. We pooled RNA from two independent experiments and used three such pooled samples as replicates for each treatment. The MicroArray Core Facility at the University of Vermont prepared the probes, hybridized the chips, and statistically analyzed the data (using the GeneSifter program). We used the Microarray Suite program to examine the data.

## Authors' contributions

LPM and TQ designed and carried out many of the experiments in cultured cells and flies; BB carried out the immuno-precipitation experiments with cultured cells and helped in interpretation of data; MLC made the Sca GFP construct and assisted in many experiments; AH performed some experiments in cultured cells and prepared and maintained cell lines; FA assisted in statistical analyses and interpretation of data; and CSW conceived the study, designed experiments, and performed or participated in many of experiments in cultured cells and flies. LPM, BB, and MLC helped CSW in drafting the manuscript. All authors read and approved the final manuscript.
